# The cGAS-STING Pathway in Hematopoiesis and Its Physiopathological Significance

**DOI:** 10.3389/fimmu.2020.573915

**Published:** 2020-11-30

**Authors:** Weinian Liao, Changhong Du, Junping Wang

**Affiliations:** State Key Laboratory of Trauma, Burns and Combined Injury, Institute of Combined Injury, Chongqing Engineering Research Center for Nanomedicine, College of Preventive Medicine, Army Medical University (Third Military Medical University), Chongqing, China

**Keywords:** cGAS-STING pathway, Hematopoiesis, Hematopoietic stem and progenitor cells, Cytosolic DNA sensing, Innate Immunity and inflammation

## Abstract

Cytosolic DNA sensing is a fundamental mechanism by which organisms handle various stresses, including infection and genotoxicity. The hematopoietic system is sensitive to stresses, and hematopoietic changes are often rapid and the first response to stresses. Based on the transcriptome database, cytosolic DNA sensing pathways are widely expressed in the hematopoietic system, and components of these pathways may be expressed at even higher levels in hematopoietic stem and progenitor cells (HSPCs) than in their certain progeny immune cells. Recent studies have described a previously unrecognized role for cytosolic DNA sensing pathways in the regulation of hematopoiesis under both homeostatic and stress conditions. In particular, the recently discovered cyclic GMP-AMP synthase (cGAS)-stimulator of interferon genes (STING) pathway is a critical modulator of hematopoiesis. Perturbation of the cGAS-STING pathway in HSPCs may be involved in the pathogenesis of hematopoietic disorders, autoimmune diseases, and inflammation-related diseases and may be candidate therapeutic targets. In this review, we focus on the recent findings of the cGAS-STING pathway in the regulation of hematopoiesis, and its physiopathological significance including its implications in diseases and therapeutic potential.

## Introduction

Hematopoietic stem and progenitor cells (HSPCs) in the bone marrow (BM) are responsible for the maintenance of both the homeostatic and stressed hematopoiesis. By modulating the balance between self-renewal and multilineage differentiation, HSPCs give rise to all mature hematopoietic cell types within the blood and immune system, including lymphoid cells, granulocytes, monocytes, dendritic cells (DCs), erythrocytes, and platelets (1). Granulocytes, monocytes, erythrocytes, and platelets are collectively called myeloid cells. Under homeostatic conditions, HSPCs are maintained in a quiescent state in the BM and undergo balanced differentiation into lymphoid and myeloid cells. However, the hematopoietic system is sensitive to stresses, such as infections and genotoxicity, and hematopoietic changes are often the rapid and the first response to stress. Under stress conditions, the quiescent state is disrupted, and HSPCs will biasedly differentiate into specific hematopoietic populations. During these processes, stresses are directly sensed by HSPCs or by HSPC progeny cells and niche cells, which in turn modulate hematopoiesis (2). Therefore, HSPCs serve as integrative hubs for the conversion of stress signals into demand-adapted hematopoiesis.

The mechanisms by which HSPCs sense stress have not been completely elucidated; however, it is well-recognized that the recognition of stress-associated molecular patterns and triggering of the immune response are fundamental functions of the immune system (3). Among these pathways, aberrant nucleic acid recognition has been regarded as a key mechanism. The pathways involved in RNA recognition have been reviewed elsewhere (4–6) and will not be covered here. Regarding DNA recognition, Toll-like receptor 9 (TLR9), absent in melanoma 2 (AIM2) and cyclic GMP-AMP synthase (cGAS), are the main specialized receptors that initiate DNA-driven immune responses in mammalian cells (7). The detection of cytosolic DNA activates the stimulator of interferon genes (STING)-dependent type I interferon (IFN-I)-stimulatory pathway, which is essential for antiviral responses. Generally, the cGAS-STING pathway is required for the IFN-I response triggered by various types of exogenous pathogenic DNA. Loss of the compartmentalization of endogenous DNA, including nuclear DNA (nDNA) and mitochondrial DNA (mtDNA), as well as disturbances in endogenous DNA metabolism also result in cytosolic self-DNA accumulation, which induces immune responses *via* the cGAS-STING pathway (8–10). Emerging evidence has indicated roles for the cGAS-STING pathway in modulating antitumor immunity (8, 9). Therefore, cellular and organismal homeostasis may be achieved through the recognition of aberrant cytosolic DNA and activation of the cGAS-STING pathway.

Interestingly, based on the transcriptome database, cGAS may be expressed at high levels in HSPCs and even exceed the levels in their certain progeny cells (11). In this review, we primarily focus on the role of the cGAS-STING pathway in hematopoietic regulation and its physiopathological significance. We envision that a better understanding of the stress sensing in HSPCs mediated by the cGAS-STING pathway will help refine potential targets for interventions in patients with hematopoiesis-related diseases.

## The cGAS-STING Pathway

**Figure 1:**
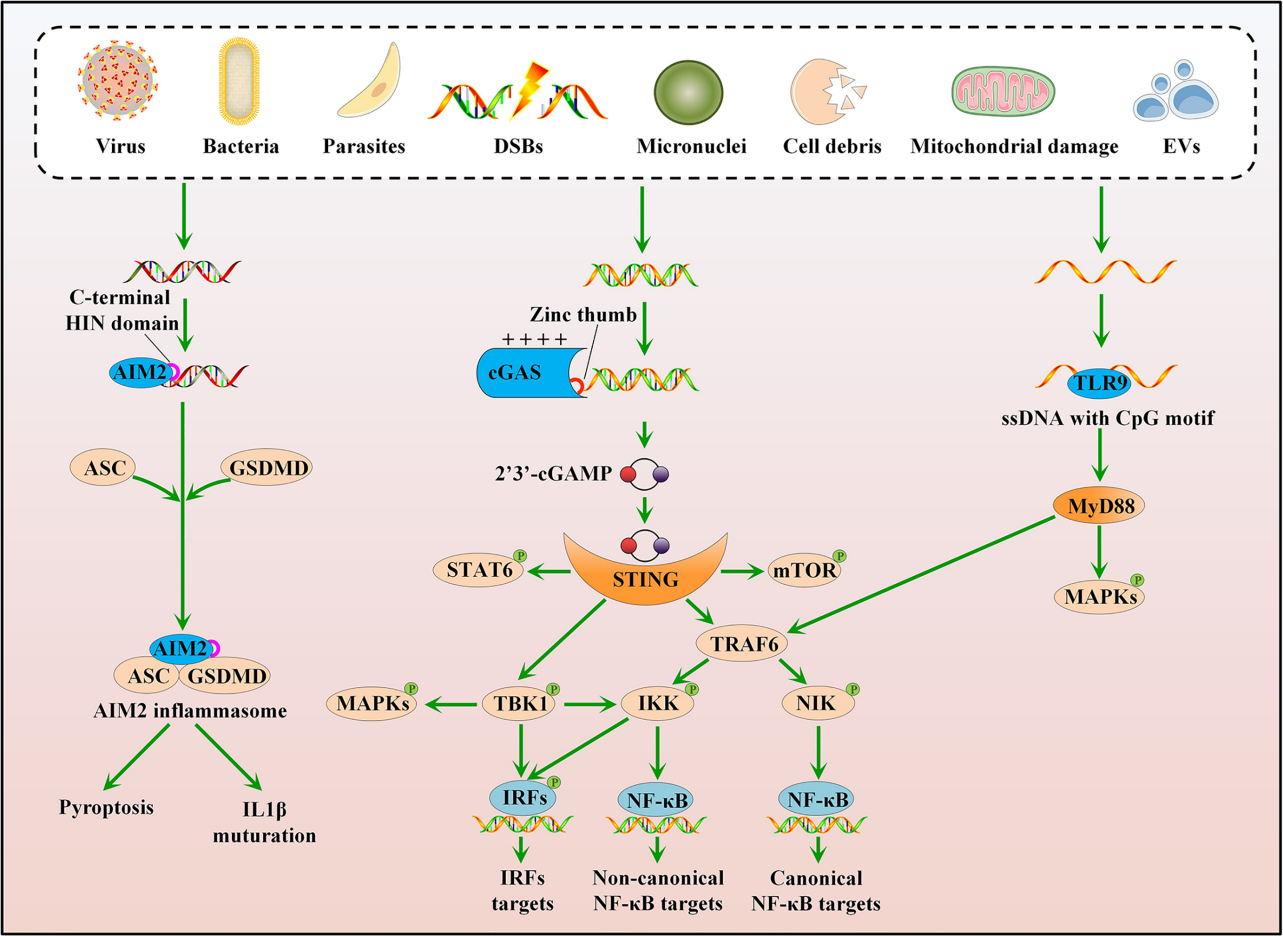
Cytosolic DNA sensing pathways. cGAS can sense various stress signals of cytosolic DNA and trigger a STING-dependent response. Upon activation, STING mainly recruits and activates three kinases: TBK1, IKK, and NIK. Besides, the cGAS-STING pathway can directly or indirectly activate IRF1, IRF5, IRF7, mTOR, STAT6, and MAPKs. TLR9 and AIM2 also recognize cytosolic DNA. The ligated TLR9 interacts with MyD88 to activate TRAF6 and MAPKs, whereas activated AIM2 associates with ASC and GSDMD to form an inflammasome complex.

cGAS is a recently discovered DNA-binding protein that condenses ATP and GTP to synthesize 2’3’-cyclic GMP-AMP (2’3’-cGAMP), which functions as a second messenger that activates STING (12) (**Figure 1**). In fact, cGAS is not a cytosolic protein but is instead localized to the plasma membrane *via* the interaction of an N-terminal phosphoinositide-binding domain with phosphatidylinositol 4,5-bisphosphate. The location of cGAS at the plasma membrane ensures the efficient discrimination between self- and non-self-DNA, as cGAS mutants defective in lipid binding induce potent IFN-I responses to genotoxic stress (self-DNA) but weaker responses to viral infection (non-self-DNA) (13). cGAS binds double-stranded DNA (dsDNA) and sometimes single-stranded DNA (ssDNA) *via* a positively charged surface and its zinc thumb to interact with the DNA (14). The activation of cGAS is also modulated by circular RNA (15), protein interactions (16–18), and posttranslational modifications, including glutamylation, ubiquitination, acetylation, and sumoylation (19–23).

Upon activation, STING translocates from the endoplasmic reticulum (ER) membrane to the Golgi and primarily recruits and activates three kinases: TANK-binding kinase 1 (TBK1), which phosphorylates interferon regulatory factor 3 (IRF3); IκB kinase (IKK), which is recruited by TNF receptor-associated factor 6 (TRAF6) and subsequently activates canonical nuclear factor-κB (NF-κB) signaling; and mitogen-activated protein kinase kinase kinase 14 (MAP3K14, best known as NIK), which activates noncanonical NF-κB signaling (**Figure 1**) (24). TBK1 acts redundantly with IKK to drive NF-κB signaling, and IKKα/IKKβ also activates IRF3 (**Figure 1**) (25, 26). The cGAS-STING pathway directly or indirectly activates other signaling molecules, such as IRF1, IRF5, IRF7, mammalian target of the rapamycin (mTOR), signal transducer and activator of transcription 6 (STAT6), and mitogen-activated protein kinases (MAPKs), including p38, extracellular signal-regulated kinases 1/2 (ERK1/2), and c-Jun-N-terminal kinase (JNK) (**Figure 1**) (27–33). Consequently, the activated cGAS-STING pathway induces the production of IFN-I and other cytokines involved in the immune response and inflammation. The cGAS-STING pathway is also implicated in the regulation of other important cellular processes, such as autophagy, cell cycle, cell death, and chromosomal stability, to eliminate pathogens, infected cells, or malignantly transformed cells (34–38).

Other sensors, including TLR9 and AIM2, also recognize cytosolic DNA. TLR9 plays a role in DNA sensing in the endosomal compartment and preferentially binds to unmethylated CpG motifs of ssDNA (39), whereas AIM2 senses cytosolic DNA *via* its C-terminal HIN domain in a sequence-independent manner (**Figure 1**) (40). Consequently, the ligated TLR9 interacts with its proximal adaptor protein, myeloid differentiation primary response 88 (MyD88), to activate TRAF6 and MAPKs (41); activated AIM2 associates with the adaptor molecule apoptosis-associated speck-like protein containing caspase recruitment domain (ASC), to form an inflammasome complex, which activates gasdermin D (GSDMD)-mediated pyroptosis and caspase 1 (CASP1)-dependent maturation of interleukin 1β (IL1β) (**Figure 1**) (40, 42). However, both TLR9 and AIM2 are dispensable for IFN-I induction, suggesting a central role of the cGAS-STING pathway in cytosolic DNA sensing.

**Figure 2:**
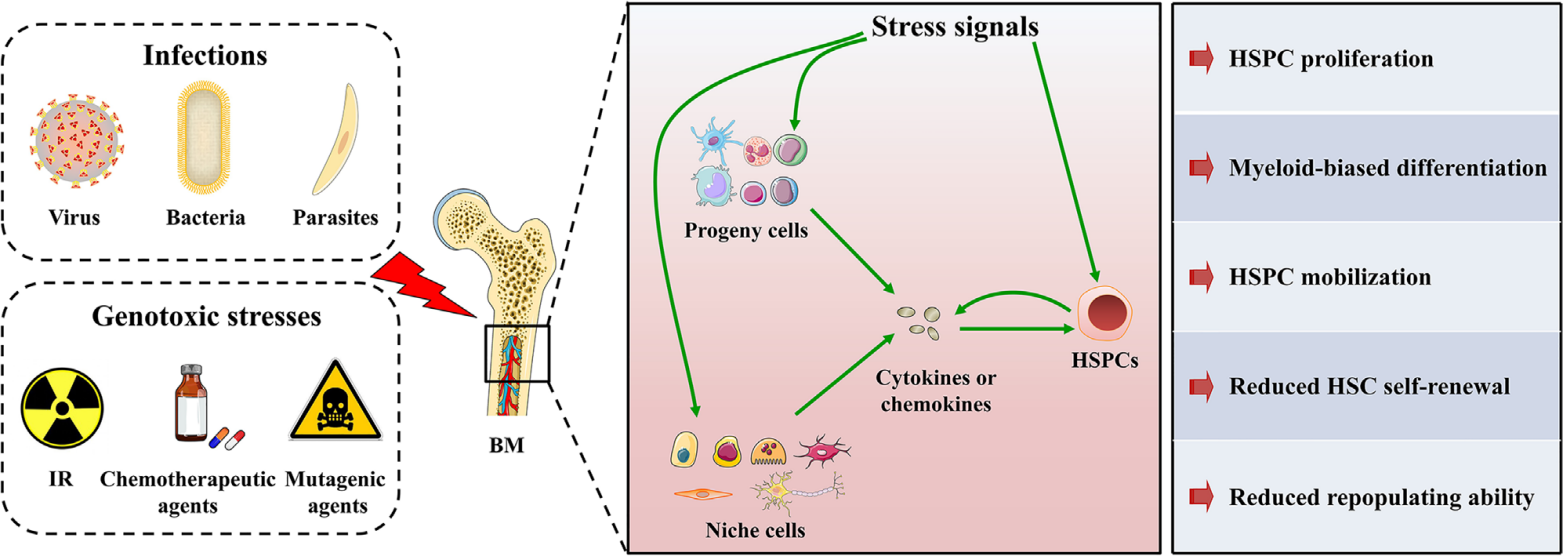
HSPC maintenance under stress conditions. HSPCs in BM are sensitive to various stresses. On one hand, HSPCs can directly sense and respond to stress signals through specialized receptors. On the other hand, HSPC progeny cells and niche cells in the BM microenvironment can sense stress signals and convert the stress signals into the secretion of cytokines or chemokines, which in turn modulate HSPC maintenance. In addition, HSPCs themselves possess cytokine production ability that even overmatches that of their progeny cells in both magnitude and breadth. Therefore, HSPCs can also convert stress signals into cytokine or chemokine signals and modulate their maintenance in an autocrine manner. Consequently, HSPCs will undergo mobilization, proliferation, and myeloid-biased differentiation to rapidly produce myeloid cells, but their self-renewal and repopulating ability will be mitigated.

Upon stresses, such as infections and genotoxicity, a considerable number of hematopoietic cells, especially myeloid cells, are depleted. The hematopoietic system needs to respond rapidly to replenish myeloid cells *via* a process called “emergency myelopoiesis” (43). According to recent studies, HSPCs may be the central hub of stress responses (43–48). HSPCs are able to skip several steps in the traditional tree-like hematopoietic hierarchy and directly differentiate into myeloid progenitor cells (49–51). Meanwhile, hematopoietic stem cells (HSCs) are heterogeneous, and myeloid-biased HSC subtypes may selectively expand in response to stress (52). Through these mechanisms, the hematopoietic system rapidly satisfies the increased demand for myeloid cells during stress. Therefore, the rapid myeloid-biased differentiation of HSPCs is required for the hematopoietic system to adapt to stresses. Although the cGAS-STING pathway is essential for sensing and defending against stresses, its role in regulation hematopoiesis has rarely been examined. The present review summarizes the role of the cGAS-STING pathway to obtain a better understanding of the mechanism regulating hematopoiesis (**Figure 2**).

## The cGAS-STING Pathway in Steady-State Hematopoiesis

A circular RNA named cia-cGAS was recently reported to be expressed at high levels in the nuclei of HSCs. Cia-cGAS displays a stronger binding affinity for cGAS than self-DNA. Cia-cGAS binds cGAS in the nucleus to block its synthase activity under steady-state condition, while a cia-cGAS deficiency will lead to increased expression of IFN-I in the BM but decreased numbers of dormant HSCs. Therefore, cia-cGAS suppresses the cGAS-mediated production of IFN-I in HSCs and the exhaustion of dormant HSCs (**Table 1**) (15). R-loops, the nucleic acid structures consisting of displaced ssDNAs and RNA : DNA hybrids, also activate cGAS-STING pathway in HSPCs. DEAD-box Helicase 41 (DDX41) binds R-loops and acts as a suppressor of R-loop accumulation. DDX41 insufficiency will result in excessive R-loop accumulation, which subsequently activate the cGAS-STING-NF-κB pathway to promote HSPC expansion *via* secreting inflammatory cytokines (**Table 1**) (53). Meanwhile, BAK/BAX-mediated apoptosis will trigger the release of mtDNA, which is recognized by the cGAS-STING pathway. The subsequent production of IFN-I in hematopoietic cells will expand HSCs but suppress their self-renewal capacity (**Table 1**). However, caspase activation following BAK/BAX activation will attenuate the IFN-I response possibly through global cellular demolition (54). In addition, gain-of-function STING mutation-induced constitutive activation of the cGAS-STING pathway causes myeloid cell expansion and lymphoid cell cytopenia in an IRF3-independent manner (75, 76). Of note, in this case, the HSC pool is only moderately affected, whether HSC function such as self-renewal capacity is influenced remains unknown (76).

**Table 1:** Roles of stress-related pathways including the cGAS-STING pathway in regulating HSPC maintenance.

Species	Context	Target	Target cells	Outcome	Mechanism	Reference
Mouse	Homeostasis	cGAS-STING pathway	HSCs	Proliferation; Reduced self-renewal	Cia-cGAS is a suppressor of cGAS. Cia-cGAS deficiency results in the hyperactivation of cGAS-STING pathway and increased production of IFN-I.	(15)
Mouse	Homeostasis	cGAS-STING pathway	HSPCs	Proliferation	DDX41 insufficiency results in excessive R-loop accumulation, which subsequently activate cGAS-STING-NF-κB pathway to promote HSPC expansion.	(53)
Mouse	Homeostasis	cGAS-STING pathway	HSCs	Proliferation; Reduced self-renewal	Apoptotic caspases deficiency triggers the release of mtDNA, which induces the constitutive activation of cGAS-STING pathway and increased IFN-I production.	(54)
Mouse	c-di-GMP administration	cGAS-STING pathway	HSPCs	Proliferation; Myeloid-biased differentiation; Mobilization; Reduced self-renewal	Bacterial c-di-GMP activates STING pathway in a cGAS-independent manner, the downstream IRF3/IFN-I signaling induces HSPC proliferation and myeloid-biased differentiation but reduces HSC self-renewal and mobilization; the downstream NF-κB/G-CSF and JNK/TGF-β promotes HSC mobilization.	(55)
Mouse	Homeostasis	IRF1/IRF2	HSCs	Proliferation; Reduced self-renewal	IRF1 is a downstream effector of both cGAS-STING pathway and IFN-I signaling. IRF2 can repress IRF1 through competitively binding to the promoter of target genes. IRF1 activation or IRF2 deficiency will promote proliferation but reduce self-renewal of HSCs.	(56)
Mouse	Genotoxic stress (IR)	IRF5	HSPCs	Proliferation; Apoptosis	IRF5 is a downstream effector of both cGAS-STING pathway and IFN-I signaling. IRF5 is upregulated and may contribute to increased proliferation, replication stress, and apoptosis of HSPC after IR.	(57)
Mouse; Zebrafish	Homeostasis	IRF7	HSCs	Myeloid-biased differentiation; Reduced HSC development	IRF7 is a downstream effector of both cGAS-STING pathway and IFN-I signaling. IRF7 activation will reduce HSC formation in the AGM region and T cell differentiation of HSCs.	(58)
Human; Mouse	Infection; Inflammation	NF-κB	HSCs	Proliferation; Myeloid-biased differentiation; Reduced self-renewal	NF-κB is a main downstream effector of both cGAS-STING pathway and cytokine signaling such as IL1 and TNF. NF-κB can induce the transcriptional activation of myeloid transcription factor PU.1.	(59–62)
Mouse	Homeostasis	TRAF6/IKK/NF-κB	HSCs	Proliferation; Myeloid-biased differentiation; Reduced self-renewal	TRAF6 or IKK deficiency-induced reduction of basal NF-κB signaling can promote proliferation and myeloid-biased differentiation but reduce self-renewal of HSCs.	(63)
Human; Mouse	Homeostasis; Stress	mTOR or MAPKs	HSCs	Proliferation; Myeloid-biased differentiation; Reduced self-renewal	mTOR or MAPKs signaling activation is associated with increased proliferation and myeloid-biased differentiation, but reduced self-renewal of HSCs.	(29, 64–66)
Mouse	pI:C administration	IFN-I signaling	HSPCs	Proliferation; Myeloid-biased differentiation	Acute IFN-I signaling activates STAT1 and Akt/mTOR to upregulate the expression of Sca-1 and myeloid markers in HSPCs. Besides, IFN-I can also upregulate IRFs in a positive feedback manner.	(55, 56, 67–69)
Mouse; Zebrafish	Homeostasis; LPS and Pam3CSK4 administration Genotoxic stress (Chemotherapeutic agent [5-FU]); Aging	IL-6 signaling	HSPCs	Proliferation; Myeloid-biased differentiation; Reduced self-renewal	IL-6 signaling may engage the Akt/mTOR and SHP2/STAT3 pathway to modulate HSPC maintenance. Besides, it is a particularly important modulator in mediating rapid myeloid cell recovery during chemotherapy-induced neutropenia.	(48, 70, 71),
Mouse	Transplantation; Inflammation	TNF signaling	HSCs	Reduced clonal growth and self-renewal	TNF can strongly inhibit the clonal growth and compromise the repopulation capacity of HSPCs that engages its two distinct receptors.	(72)
Human; Mouse	Infection; Inflammation	NF-κB	HSPCs	Reduced necroptosis	NF-κB can induce the transcriptional activation of cIAP2, which can inhibit RIPK3/MLKL-mediated necroptosis.	(62)
Human; Mouse	Infection; DNA introduction; pI:C administration	IFN-I signaling	HSCs	Inhibited proliferation; Apoptosis; Necroptosis	Chronic IFN-I signaling may inhibit HSC proliferation and trigger p53 and RIPK1-CASP8 pathway-mediated apoptosis, or RIPK3- and MLKL-mediated necroptosis of HSCs.	(36, 73, 74),
Mouse	pI:C administration	IFN-I signaling	HSCs	DNA damage	IFN-I signaling induces mitochondrial ROS overproduction and causes DNA damage in HSCs.	(46)

## Regulation of HSPC Maintenance by the cGAS-STING Pathway

The cGAS-STING pathway directly and indirectly regulates cell activities, including proliferation, differentiation, cell death, autophagy, senescence, and metabolism, through the activation of its downstream effectors or the secretion of cytokines. However, evidence for whether and how this pathway regulates HSPC maintenance is scarce. We discuss the recently discovered functions of the cGAS-STING pathway and its potential roles in regulating HSPC maintenance.

Downstream effectors of the cGAS-STING pathway, including IRFs, NF-κB, mTOR, and MAPKs, are closely associated with hematopoiesis. The roles of mTOR and MAPKs in HSPC maintenance are well-defined (**Table 1**) (29, 64–66). IRFs and NF-κB are transcription factors, and their activation directly modulates hematopoiesis at the genetic level. IRF1, IRF3, IRF5, and IRF7 act downstream of the cGAS-STING pathway (31–33, 55, 77). IRF2 represses IRF1 by competitively binding to the promoter of target genes. IRF1 activation and IRF2 deficiency promote the proliferation of HSCs but reduce their self-renewal capacity (**Table 1**) (56). IRF3 is implicated in infection-associated HSPC proliferation and a reduction in self-renewal (55). IRF5 may participate in the mechanism regulating HSPC survival, because a deletion of IRF5 protects HSPCs from DNA damage-induced apoptosis (**Table 1**) (57). IRF7 activation reduces HSC populations in the aorta-gonad-mesonephros (AGM) region and the T cell differentiation of HSCs, suggesting roles for IRF7 in HSC development and specification (**Table 1**) (58). These data suggest distinct functions of IRFs in regulating HSC maintenance. NF-κB promotes the myeloid differentiation of HSCs through the transcriptional activation of the myeloid transcription factor PU.1 (**Table 1**) (59). However, a reduction in basal NF-κB signaling induced by a TRAF6 or IKK deficiency promotes proliferation and myeloid-biased differentiation but reduces the self-renewal of HSCs under homeostatic conditions (**Table 1**) (63).

The most important function of the cGAS-STING pathway is the production of various cytokines and chemokines. HSPCs and their progeny and niche cells contribute to cytokine production during stress. Notably, the ability of HSPCs to produce cytokines exceeds their progeny cells in both magnitude and breadth (48). Given the high expression of cytokine and chemokine receptors, cytokines and chemokines may help HSPCs rapidly adapt to stresses in an autocrine manner. Here, we discuss the most common cytokines downstream of the cGAS-STING pathway, including IFN-I, Interleukin-1 (IL-1), IL-6, and TNF.

IFN-I is the hallmark cytokine produced following the activation of the cGAS-STING pathway. Acute IFN-I signaling promotes HSC proliferation but reduces the self-renewal capacity and mobilization of HSCs (**Table 1**) (55, 56, 67, 68). IFN-I signaling increases the phosphorylation of STAT1 and Akt/mTOR and upregulates the expression of stem cell antigen-1 (Sca-1) and myeloid markers, such as CD41. Conversely, inhibition of STAT1, Akt/mTOR, or Sca-1 will cause HSCs to become insensitive to IFN-I stimulation, indicating that STAT1, Akt/mTOR, and Sca-1 mediate the effects of IFN-I (67, 68). IFN-I also upregulates IRFs through a positive feedback mechanism (69), which may in turn regulate HSPC maintenance, as described above. However, chronic IFN-I signaling inhibits HSC proliferation and triggers apoptosis mediated by p53 and the receptor-interacting protein kinase-1 (RIPK1)-CASP8 pathway-mediated or RIPK3- and mixed-lineage kinase domain-like protein (MLKL)-mediated necroptosis of HSCs, leading to HSC exhaustion (**Table 1**) (36, 73, 74, 78). Notably, IFN-I signaling-induced mitochondrial reactive oxygen species (ROS) overproduction causes DNA damage in HSCs (**Table 1**) (46), which provides a novel connection between stress hematopoiesis and the occurrence of DNA damage and decreased function of adult HSCs.

IL-1 promotes the proliferation and myeloid-biased differentiation of HSCs and prevents chemotherapy-mediated myelosuppression by activating the NF-κB-PU.1 axis, but it mitigates the self-renewal capacity of HSCs (**Table 1**) (59–61). Similarly, IL-6 also promotes HSPC proliferation and myeloid-biased differentiation in an auto- or paracrine manner (**Table 1**) (48, 70, 71). In particular, Zhao et al. identified IL-6 as a particularly important modulator that mediates rapid myeloid cell recovery during chemotherapy-induced neutropenia (48).

TNF overproduction is closely associated with the BM hematopoietic failure observed in many patients with acute or chronic inflammatory diseases. TNF strongly inhibits clonal growth and compromises the repopulation capacity of HSCs (72). Notably, actively cycling rather than quiescent HSCs are the primary targets of TNF-mediated hematopoietic suppression (**Table 1**) (72). The latest research has revealed different effects of TNF on HSCs, and myeloid progenitors are actually different. TNF prevents HSC necroptosis and promotes myeloid-biased differentiation *via* the NF-κB-dependent transcriptional activation of cellular inhibitor of apoptosis protein 2 (cIAP2) and PU.1, respectively, whereas it induces myeloid progenitor apoptosis. Consequently, TNF-α prevents the necroptosis, but not apoptosis, of HSPCs and poises HSPCs for myeloid cell production but kills other blood cells, which facilitates hematopoietic clearance and promotes regeneration in response to inflammatory stress (**Table 1**) (62).

## The cGAS-STING Pathway in Stressed Hematopoiesis

### Infection

The cGAS-STING pathway protects against infections with viruses, bacteria, and protozoan parasites by sensing pathogenic DNA, infection-induced mitochondrial damage, bacterial proteins, virulence factors and metabolites, and bacteria-derived cyclic dinucleotides (CDNs) (79–84). Meanwhile, infected cells transactivate the cGAS-STING pathway in bystander cells *via* the cell-to-cell transmission of cGAMP or the release of extracellular vesicles (EVs) (85–89). Based on accumulating evidence, HSPCs, and not only their progeny immune cells, are able to directly sense and respond to infections. For example, HSPCs directly sense a mimic of viral nucleic acid, polyinosinic:polycytidylic acid (pI:C), to elicit IFN-I production through a process independent of TLR signaling (68). Meanwhile, HSPCs were recently shown to sense one of the CDNs, c-di-GMP, *via* STING. Upon c-di-GMP exposure, STING activation in HSPCs induces HSC entry into the cell cycle and promotes HSPC proliferation and mobilization, but it decreases the number and repopulation capacity of LT-HSC (55). On the other hand, HSPCs indirectly recognize the acute or chronic infectious state by sensing the extrinsic signals, such as the cytokines or chemokines released by their progeny immune cells, niche cells, or themselves (**Figure 2**). In particular, the abundance of these receptors on HSPCs increases significantly during systemic infections (48).

### Genotoxic Stress

Genotoxic stress is derived from a wide variety of physical and chemical sources, such as ionizing radiation, chemotherapeutic agents, and mutagenic agents. In fact, the long-living property of HSCs makes these cells particularly susceptible to genotoxic stress. HSCs are thought to be the gatekeeper of genomic integrity of the entire hematopoietic system, because an unrepaired genetic lesion in a single HSC may spread throughout the HSC pool and propagate to all of the hematological compartments *via* self-renewing division and differentiation (90). DNA damage occurs when cells are exposed to genotoxic stress. In fact, HSPCs are sensitive to genotoxic stress-induced DNA damage, including DSBs and micronuclei formation (91). Recent advances suggest that the cGAS-STING pathway may play an essential role in the genotoxic stress of HSPCs by sensing DSBs and micronuclei (91–94). Therefore, reacquainting the role of the cGAS-STING pathway in HSPC (**Table 1**) maintenance under genotoxic stress is of great value to understand the pathogenesis of some hematopoietic disorders.

## Outlook of the cGAS-STING Pathway in HSPC

Numerous functions of the cGAS-STING pathway have been established recently, and many of these functions may be extrapolated to HSPC maintenance. First, the activation of the cGAS-STING pathway may promote or inhibit HSC death, depending on the context or HSC subtype. Activation of the cGAS-STING pathway in mouse myeloid cells allows them to resist apoptosis, but activation is amplified in mouse lymphoid cells and results in apoptosis (95). Activation of the cGAS-STING pathway in human myeloid cells also elicits lysosomal cell death with apoptotic-like features (35). Besides, activation of cGAS-STING pathway induces apoptosis by disrupting calcium homeostasis and/or suppressing of B-cell lymphoma-extra large (Bcl-xL) expression (17). A positive feedback loop of death signaling may also exist because PUMA induction during cell death may promote the cytosolic release of mtDNA and subsequent activation of the cGAS-STING pathway (96). Autophagy, which is closely associated with HSC maintenance (97), is a primordial function of the cGAS-STING pathway. The cGAS-STING pathway induces autophagy by facilitating the lipidation of microtubule-associated protein 1 light chain 3 (LC3) or inactivating mTOR. However, the cytosolic chromatin fragments that result from replicative crisis-induced telomeric DNA damage activate the cGAS-STING pathway and engage the autophagic cell death to restrict chromosomal instability and prevent tumorigenesis (98). cGAS is also recruited to DSBs in the nucleus and interacts with poly(ADP-ribose) polymerase 1 (PARP1) to suppress homologous recombination by impeding the formation of the PARP1-Timeless complex, which may aggravate genomic instability and subsequently induce malignant transformation or cell death (99, 100).

## Translational Implications

An understanding of the role of the cGAS-STING pathway in regulating hematopoiesis provides new insights into the pathogenesis of and interventions for hematopoietic disorders. Gain-of-function mutations or aberrant activation of the cGAS-STING pathway may participate in the pathogenesis of myelopoiesis-associated clonal hematopoiesis and myeloid malignancies (75, 101, 102). Recent advances spotlighted on the role of leukemia stem cells (LSCs) in the driving of persistent disease and the relapse after robust antitumor therapy (103). Given the many common characteristics shared between LSCs and HSCs, aberrant activation of the cGAS-STING pathway may be involved in the maintenance of myeloid LSCs through promoting malignant reprogramming or self-renewal (104, 105). On the other hand, the activation of a cGAS-STING pathway may improve the therapeutic effect of myeloid leukemia *via* inducing cell death or differentiation of myeloid LSCs (95, 106, 107).

The cGAS-STING pathway in HSPCs may be employed as a therapeutic target or to develop vaccine adjuvants for chronic infections. Activation of the cGAS-STING pathway may promote the myeloid-biased differentiation of HSPCs to enhance the anti-infection reservoir of the immune system. However, it should bear in mind that the activation of the cGAS-STING pathway may also increase the vulnerability to or severity of infections through dampening adaptive immunity, exacerbating inflammation or facilitating pathogen replication, survival, and infection (81, 108–113).

HSPC maintenance is closely associated with the pathogenesis of autoimmune diseases. HSPCs confer protection to secondary infection, in a phenomenon termed “trained innate immunity” or “innate immune memory,” which contributes to autoimmune diseases through exaggerating immune responses (114–116). Meanwhile, the myeloid progenitors released from the BM may infiltrate peripheral tissues and produce primed neutrophils *in situ* to exacerbate inflammatory responses within the affected tissues (117, 118). On the other hand, patients with autoimmune diseases may have a poor response to infectious challenges due to the deficiency of lymphoid cells (119). During these processes, the cGAS-STING pathway in HSPCs may play an important role.

Myelopoiesis or lymphocytopenia are closely associated with inflammation-related diseases including gain-of-function STING mutation-induced vasculopathy, cardiovascular diseases (CVD), and metabolic diseases (75, 120–126). Conversely, the modulation of HSPC maintenance that reduces myeloid cell production can protect against CVD (127, 128). Therefore, the cGAS-STING pathway in HSPCs may be a promising target to provide new insights into the pathogenesis and treatment of inflammation-related diseases.

The cGAS-STING pathway has been considered as a promising immunotherapy target in recent years (8). Synergetic effects can be achieved in multiple tumors using the combination immunotherapies with cGAS-STING pathway agonists (129, 130). However, the activated cGAS-STING pathway may promote growth or metastasis of certain tumors (131), and its chronic activation paradoxically induces an immune-suppressive tumor microenvironment by promoting the myeloid-biased differentiation of HSPCs and apoptosis of T cells (95). Individualized or specific cell-targeted application of cGAS-STING pathway agonists or antagonists will be feasible in the future.

## Conclusions

Although the immunomodulatory effects of the cGAS-STING pathway in hematopoietic cells have been extensively studies, little attention has been paid to its roles in hematopoiesis. The regulation of HSPC maintenance is an attractive field to understand hematopoiesis under homeostatic and stress conditions. Components of the cGAS-STING pathway may be expressed at high levels in HSPCs, which may allow HSPCs to rapidly sense and respond to stresses, including infection and genotoxicity, by promoting HSPC proliferation, mobilization, and myeloid-biased differentiation (**Figure 2**). Moreover, this rapid response may also facilitate the elimination of damaged HSPCs or malignantly transformed HSPCs *via* the induction of cell death to maintain the fitness of the HSPC pool. On the other hand, modulation of the cGAS-STING pathway in HSPCs may boost anti-infection or antitumor immunity and suppress autoimmunity and peripheral inflammation. However, multiple obstacles must be addressed before its clinical application. For example, chronic activation of the cGAS-STING pathway may promote the pathogenesis of myeloid malignancies or induce an adaptive immunodeficiency. Future studies aiming to discover the distinct functions of the cGAS-STING pathway in HSPC maintenance and develop modulators targeting specific cell types will be meaningful.

## Author Contributions

CD and JW decided on the topics. WL and CD wrote the manuscript and prepared the figures and the table. All authors contributed to the article and approved the submitted version.

## Funding

This work was supported by the Natural Science Foundation of China (No. 81725019, 81930090, 81874256).

## Conflict of Interest

The authors declare that the research was conducted in the absence of any commercial or financial relationships that could be construed as a potential conflict of interest.

## Abbreviations

AIM2 absent in melanoma 2 CASP1 caspase 1; CDNs: cyclic dinucleotides; cGAS: cyclic GMP-AMP synthase; CMPs: common myeloid progenitors; DCs: dendritic cells; DDR: DNA damage response; GMPs: granulocyte-monocyte progenitors; GSDMD: gasdermin D; HSCs: hematopoietic stem cells; HSPCs: hematopoietic stem and progenitor cells; IFN-I: type I interferon; IL1β: interleukin 1β; IKK: IkB kinase; JNK: c-Jun-N-terminal kinase; MDS: myelodysplastic syndromes; MDPs: monocyte-dendritic cell progenitors; MyD88: myeloid differentiation primary response 88; NF-κB: nuclear factor-κB; NLRP3: NOD-, LRR-, and pyrin domain-containing protein 3; STING: stimulator of interferon genes; TBK1: TANK-binding kinase 1; TLR9: Toll-like receptor 9; TRAF6: TNF receptor associated factor 6; PARP1: poly (ADP–ribose):;: polymerase 1; PUMA: p53 upregulated modulator of apoptosis.
